# Genome-wide analysis of ionotropic receptors provides insight into their evolution in *Heliconius* butterflies

**DOI:** 10.1186/s12864-016-2572-y

**Published:** 2016-03-22

**Authors:** Bas van Schooten, Chris D. Jiggins, Adriana D. Briscoe, Riccardo Papa

**Affiliations:** Department of Biology, University of Puerto Rico, Rio Piedras, San Juan Puerto Rico; Department of Zoology, University of Cambridge, Cambridge, UK; Department of Ecology and Evolutionary Biology, University of California, Irvine, CA 92697 USA

**Keywords:** Chemosensory receptors, Speciation, Butterfly, Lepidoptera, Smell, Taste, Olfaction, Gustation

## Abstract

**Background:**

In a world of chemical cues, smell and taste are essential senses for survival. Here we focused on *Heliconius*, a diverse group of butterflies that exhibit variation in pre- and post-zygotic isolation and chemically-mediated behaviors across their phylogeny. Our study examined the ionotropic receptors, a recently discovered class of receptors that are some of the most ancient chemical receptors*.*

**Results:**

We found more ionotropic receptors in *Heliconius* (31) than in *Bombyx mori* (25) or in *Danaus plexippus* (27). Sixteen genes in Lepidoptera were not present in Diptera. Only *IR7d4* was exclusively found in butterflies and two expansions of *IR60a* were exclusive to *Heliconius*. A genome-wide comparison between 11 *Heliconius* species revealed instances of pseudogenization, gene gain, and signatures of positive selection across the phylogeny. *IR60a2b* and *IR60a2d* are unique to the *H. melpomene*, *H. cydno,* and *H. timareta* clade, a group where chemosensing is likely involved in pre-zygotic isolation. *IR60a2b* also displayed copy number variations (CNVs) in distinct populations of *H. melpomene* and was the only gene significantly higher expressed in legs and mouthparts than in antennae, which suggests a gustatory function. d*N*/d*S* analysis suggests more frequent positive selection in some intronless *IR* genes and in particular in the *sara/sapho* and *melpomene/cydno/timareta* clades. *IR60a1* was the only gene with an elevated d*N*/d*S* along a major phylogenetic branch associated with pupal mating. Only *IR93a* was differentially expressed between sexes.

**Conclusions:**

All together these data make *Heliconius* butterflies one of the very few insects outside *Drosophila* where IRs have been characterized in detail. Our work outlines a dynamic pattern of IR gene evolution throughout the *Heliconius* radiation which could be the result of selective pressure to find potential mates or host-plants.

**Electronic supplementary material:**

The online version of this article (doi:10.1186/s12864-016-2572-y) contains supplementary material, which is available to authorized users.

## Background

Animals utilize a variety of cues to make key decisions over a range of behaviors, including navigating [1, 2], foraging [3], avoiding predators [4], kin recognition [5], and mate choice [6, 7]. Among the different signals, all organisms from bacteria to mammals utilize chemical cues. Chemosensory receptors (CRs) are the molecular tools that animals have evolved to distinguish a myriad of odors and tastes. This complexity in chemical sensing is manifested by the high degree of inter- and intraspecific variation at CR loci which is partly explained by the adaptation of organisms to different environments. Some of this variation is also due to genetic drift, and the random processes of gene duplication and deletion, which can generate pseudogenes and copy number variations (CNVs). Thus, given the co-founding effect of selection and genetic drift, explaining the mechanisms that underlie differences in CRs between and within species is not a simple task. To date, only a few species have been studied and our understanding of the biochemical basis of chemo-sensation is limited.

Only with massive parallel sequencing have a variety of genomic projects begun to investigate the evolution of chemosensory genes across different levels of taxonomic organization [8–18]. These studies have shown that chemosensory receptor genes and pseudogenes vary enormously among different animal species. For example, in tetrapods the olfactory receptor (OR) genes range from 400 to 2,100 depending on the species, with 20–50 % described as pseudogenes [19]. These receptors are the most studied chemosensory genes, especially in mammals where ORs have been estimated to represent around 3 % of all the genes in the genome and are thus the largest mammalian gene family known to date [20]. However, compared to mammals, insects display a more compact and simpler gene repertoire but with similar chemo-sensing functions [21]. The insect chemosensory receptors, which have evolved independently from mammals, consist mainly of gustatory receptors (GRs), olfactory receptors (ORs), and ionotropic receptors (IRs). GRs are involved in tasting sweet and bitter compounds [22] but also act as CO_2_ sensors [23] and are thought to be involved in heat avoidance [24]. The expression of GRs occurs mostly in tissues directly in contact with food or other objects being tasted. ORs are the starting point for odor coding and are expressed in olfactory organs such as antennae. IRs are the most primitive class of receptors and are expressed both in olfactory and gustatory tissues [16, 25]. Their ability to detect a wide array of solid and volatile molecules probably has to do with their ancestral role in the early protostomes [26], and the necessity to cover functions that during the evolution of chemo-sensation were reassigned to GRs and ORs.

The recent discovery and annotation of the ionotropic receptors in *Drosophila* [16, 27] has provided the opportunity to gain novel insight into the genetic and molecular basis of smell and taste in insects. Unlike mammals, insects possess IRs, which have most likely evolved from ionotropic glutamate receptors (iGluRs), a conserved family of synaptic ligand-gated ion channels [26]. IRs have been implicated in detection of phagostimulants [25], pheromones [25], salt [28], volatiles [16, 26, 27] and also seem to be involved in hearing [29]. IRs have mostly been studied in *Drosophila,* in which they form two groups, the olfaction oriented “antennal” IRs and the gustatory, mostly intronless, “divergent” IRs [25, 26]. The “antennal” IRs display higher sequence conservation, lower dN/dS ratios, fewer duplications, and fewer pseudogenes than the “divergent” intronless IRs. The divergent IR genes, also display a pattern of rapid gain and loss between species [26]. However, to understand whether the patterns of evolution in *Drosophila* IRs are similar to the patterns in other insects, additional data are needed across the insect phylogeny. The increasing availability of insect genomes offers the opportunity to better understand patterns of chemosensory gene evolution at a broader scale.

*Heliconius* butterflies are a group of insects where chemical cues have likely played a critical role in their evolution, adaptation, and speciation [30–32]. *Heliconius* butterflies have extraordinary phenotypic diversity and complex behaviors. Composed of almost 50 species, which represent a continuum of taxa across the stages of speciation, *Heliconius* includes distantly-related species with identical wing color patterns that are sexually incompatible and also closely-related interbreeding species with different wing color patterns. These incomplete species boundaries are best represented by *H. melpomene, H. cydno,* and *H. timareta,* which are closely related species that occasionally exchange genes while showing strong assortative mating [33–35]. It is known that strong mating preference is partially linked to the same genes controlling wing color pattern variation [36]. However, chemosensory genes likely play an important role in pre- and post-zygotic isolation. Indeed, smell and taste are strongly involved in insect prezygotic isolation [6, 7], host plant choice [37], and food recognition [38]. *Heliconius* butterflies, for example, show different degrees of host plant specialization, with species that are generalists and species that are specialists on one or a few species of *Passiflora* [39].

Previous studies of chemosensory receptors in *Heliconius* butterflies have revealed an unexpected diversity in ORs and GRs [34, 37], and suggested a link between the evolution of GRs and female oviposition behavior [37]. However, to date *Heliconius* IRs have not yet been examined. *Bombyx mori* [16, 40, 41] and *Danaus plexippus* [1, 16] are the only two representatives of 180,000 species of Lepidoptera for which the GR, OR and IR genes have been annotated. Here, we characterize the IR genes in *Heliconius* and conduct a comparative analysis with *B. mori* and *D. plexippus*.

## Methods

### Annotation of ionotropic receptors in *H. melpomene* and comparative analysis with Lepidoptera and Diptera

IR gene annotation was performed by TBLASTX [42] searches of IR and related iGluR genes of *Bombyx mori* [26], *Drosophila melanogaster* [26] and *Danaus plexippus* [1] against a Trinity [43] assembled RNA-Seq library of *Heliconius melpomene rosina* males (*n* = 3) and females (*n* = 3). The RNA-Seq data is deposited in the European nucleotide archive (ERP002272) [37]**.** The contigs from the Trinity assembly were aligned against the *H. melpomene* genome (v1.1) [34] in Mega 6.0 [44] using MUSCLE [45]. This resulted in almost complete genes including exon-intron boundaries and a physical location in the genome. When RNA-Seq data did not recover the whole IR gene, missing parts were identified by aligning homologues of reference species against the *H. melpomene* genome. All genes identified in *H. melpomene* as IRs were clear homologs of genes identified by Croset et al. [26] as ionotropic receptors in *Danaus plexippus* and *Bombyx mori*. For 15 *H. melpomene* genes, when using TBLASTX, the closest homolog had an E-value of 0, while the lowest of the remaining 16 was a *Heliconius-*specific duplication, *IR60a2c,* with an E-value of 1E-97. We used the gene models constructed from *H. melpomene* to improve IR annotations in *B. mori* (v2.0*)* and *D. plexippus (*v3*)* and discover unannotated genes in those genomes. The *H. melpomene* IR genes were aligned against the genomes of *D. plexippus* or *B. mori,* and if applicable the IR sequences of Croset et al. [26]. As a result of our deep RNA sequencing and annotation in *H. melpomene* we were able to improve the IR gene models significantly in all the three butterflies genomes.

*H. melpomene* IRs were named after their closest homologs described in *B. mori* or *D. plexippus* [26]. Gene phylogenies were constructed using MAFFT [46] and RAxML [47] (best of 200 trees, 500 bootstraps). We used the *H. melpomene* genome 1.1 [34] to precisely locate each IR gene across the 21 linkage groups (see Additional file 1).

### Ionotropic receptor evolution within the genus *Heliconius*

We assembled 10 *Heliconius* genomes (*H. cydno chioneus, H. timareta timareta, H. wallacei flavescens, H. hecuba, H. doris doris, H. clysonymus tabaconas, H. telesiphe sotericus, H. erato petiverana, H. sara magdalena* and *H. sapho sapho)* using ABySS v1.5.2 [48] (command: abyss-pe *n* = 5 k = 31 c = 2) as previously described by Briscoe et al. [37]*.* We used TBLASTX and Tophat [49] to identify the homolgous *H. melpomene* IRs in each species. Depending on the species we were able to obtain complete or nearly complete IR gene sequences (*n* = 282, mean of 97.9 % of bases complete).

### Detecting positive selection in IRs across the *Heliconius* adaptive radiation

The IRs annotated in the 11 *Heliconius* species and the out-group *D. plexippus* were stripped of their terminal stop codon and aligned in the correct reading frame using MEGA 6 [44] with the aligner MUSCLE [45]. Alignments were analyzed in Datamonkey [50] using the HyPhy branch-site Random Effects Likelihood (REL) model [51] to test if a branch shows signs of positive diversifying selection. In branch-site REL, Ω (the dN/dS ratio) is modeled as three variables, Ω_1,_ Ω_2_ and Ω_3._ While Ω_1_ and Ω_2_ remain between zero and one, with Ω_2_ bigger than Ω_1_, the value of Ω_3_ ≥1. Ω_3_ is the estimate of positive selection. All Ω parameters are estimated by maximum likelihood. The likelihood ratio test (LRT) test determines if Ω_3_ is bigger than one with a *p*-value corrected for multiple testing*.* The HyPhy branch-site random effects likelihood method [51] produces its own neighbor joining trees, and when the neighbor joining gene tree and the species tree from Kozak et al. [52] were not identical, the resulting branch under positive selection does not exist on the species tree of Kozak et al. [52] and is shown as a dotted line. In the case of a gene with many duplications, such as *IR60a2* (*a, b, c, d* and *e*), only the closest copy to the orthologous gene in the out-group species *D. plexippus* was utilized.

### Copy number variation in natural populations of *H. melpomene* and *H. cydno*

We tested IR Copy Number Variations (CNVs) by utilizing twenty previously published whole genome sequences [37, 53]*,* from distinct natural population of *H. melpomene* and *H. cydno*. More specifically, we analyzed four individuals for the following populations: *H. melpomene amaryllis, H. melpomene aglaope, H. melpomene rosina, H. cydno chioneus,* and *H. melpomene melpomene*. We first aligned the Illumina re-sequenced genomes against the *H. melpomene* reference genome (v1.1) [34] and then analyzed the read depth for each IR gene using CNVnator (100 bp sliding window) [54]. The output of CNVnator was used to determine candidate variable insertions and deletions. We considered estimated copy number of >2 as a potential duplication and <0.5 as a potential deletion. To control for possible artifacts in the *H. melpomene* genome assembly (v1.1) we also ran CNVnator on the raw reads used to create the reference genome and identified false positives then discarded those from further analyses.

### Ionotropic receptor expression in sensory tissues of male and female individuals of *H. melpomene*

As mentioned above, we used Illumina RNA-sequencing data (European nucleotide archive ERP002272) [37], collected from legs, mouthparts and antennae of three males and three females (two paired-end runs for every sample and three additional single-end 100 bp run for one male and one female on a HiSeq 2000 for a total of 730,617,415 reads, ~33900× coverage). Raw data was trimmed (adapter sequence, distorted 10 bp at beginning and sliding window:4:20) using Trimmomatic [55] and assembled using Trinity [43]. A differential expression analyses was done on the entire transcriptome using Trinity scripts built around the edgeR package from Bioconductor, which uses the trimmed mean of M values (TMM) normalization method [56–59]. Genes were called differentially expressed when the FDR <0.05. We calculated tissue- and sex-specific expression, and used FPKM value to generate a combined heat map for each biological replicate as well as for individual specimens. Because a Trinity assembly is *de novo*, only genes expressed high enough can be assembled and analyzed for expression. *IR60a1a* and *IR60a2b* were expressed sufficiently but *IR60a1b* and *IR60a2* (*a, c, d* and *e*) were not and were therefore omitted from analysis.

## Results

### Annotation of ionotropic receptors in the *Heliconius melpome*ne genome

**Fig. 1:**
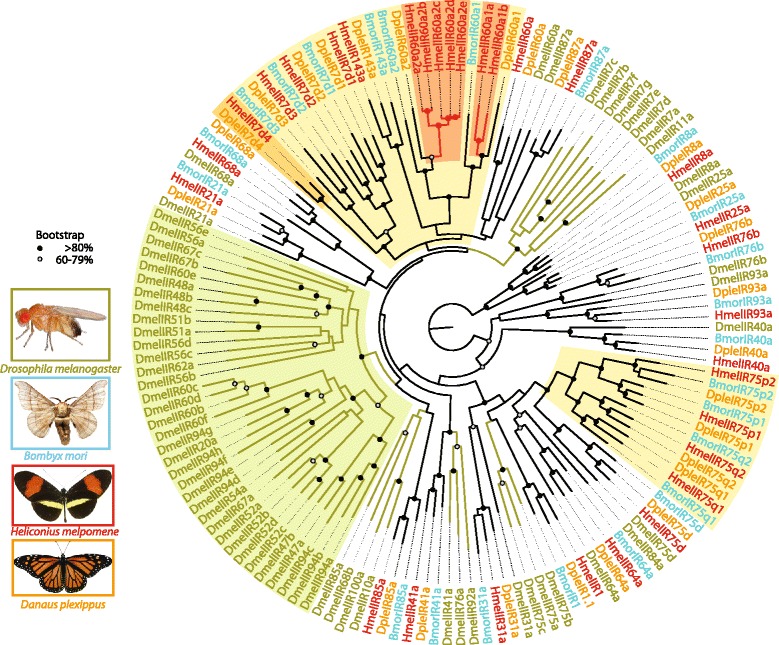
Phylogenetic relationships of ionotropic receptors in Lepidoptera and Diptera. Black dots indicate >80 % bootstrap support, grey dots indicated 60–79 % bootstrap support. Shaded colors represent taxon-specific IR genes: Diptera-specific (*green lines*), IR20a clade (*green shade*), Lepidoptera-specific (*yellow*), butterfly-specific (*orange*), and *Heliconius-*specific (*red*). While a large number of genes are conserved between Lepidoptera and Diptera, instances of lepidopteran-, butterfly- and *Heliconius*-specific IRs emerged from our analysis

Overall, with our annotation (Additional files 1 & 2) we have generated a complete description of 31 IR genes in *H. melpomene.* This is more than *B. mori* (25) and *D. plexippus* (27), but around half the number found in *Drosophila melanogaster* (66) and other Diptera [26] (Table 1, Fig. 1). We also significantly improved the gene models of most of the previously described ionotropic receptors genes in *B. mori* (*IR68a, IR7d1*, *IR7d2, IR7d3, IR8a, IR21a, IR40a, IR41a, IR75d, IR75p2, IR75q1, IR75q2, IR76b, IR87a, IR93a* and *IR143a*) and *D. plexippus* (*IR8a, IR21a, IR25a, IR31a, IR40a, IR41a, IR60a2, IR64a, IR68a, IR75p1, IR75p2, IR75q1, IR75q2, IR76b, IR87a and IR93a*)*.* Moreover we identified new putative IRs in *B. mori* and in *D. plexippus*: seven in *B. mori* (*IR1, IR31a, IR60a2, IR60a1, IR75p1, IR75p2pseudo,* and *IR85a*) (Table 1), and eleven in *D. plexippus (IR7d1, IR7d2, IR7d3, IR7d4, IR60a, IR85a* and *IR143a*) including four genes (*IR75p1, IR75p2, IR75q1 and IR75q2*) which were reported as two gene models by Zhan et al. [1] (Table 1, & Additional file 2).

**Table 1:** IR gene diversity in *H. melpomene* compared to *B. mori* or *D. plexippus*

*H. melpomene IR*	*B. mori*	*D. plexippus*
*HmIR1*	discovered	unchanged
*HmIR7d1*	improved	discovered
*HmIR7d2*	improved	discovered
*HmIR7d3*	improved	discovered
*HmIR7d4*	absent	discovered
*HmIR8a*	improved	improved
*HmIR21a*	improved	improved
*HmIR25a*	unchanged	improved
*HmIR31a*	discovered	improved
*HmIR40a*	improved	improved
*HmIR41a*	improved	improved
*HmIR60a*	absent	discovered
*HmIR60a1a*	discovered	unchanged
*HmIR60a1b*	absent	absent
*HmIR60a2a*	discovered	improved
*HmIR60a2b*	absent	absent
*HmIR60a2c*	absent	absent
*HmIR60a2d*	absent	absent
*HmIR60a2e*	absent	absent
*HmIR64a*	unchanged	improved
*HmIR68a*	improved	improved
*HmIR75d*	improved	unchanged
*HmIR75p1*	discovered	improved
*HmIR75p2*	improved & discovered pseudogene	improved
*HmIR75q1*	improved	improved
*HmIR75q2*	improved	improved
*HmIR76b*	improved	improved
*HmIR85a*	discovered	discovered
*HmIR87a*	improved	improved
*HmIR93a*	improved	improved
*HmIR143a*	improved	discovered

The list of homologous IR genes between the three Lepidoptera genomes is reported relative to the IRs in *H. melpomene*. Information is also provided for each gene if its sequence was either left unchanged, improved, or discovered in our study when compared to data from Croset et al. [26]

**Fig. 2:**
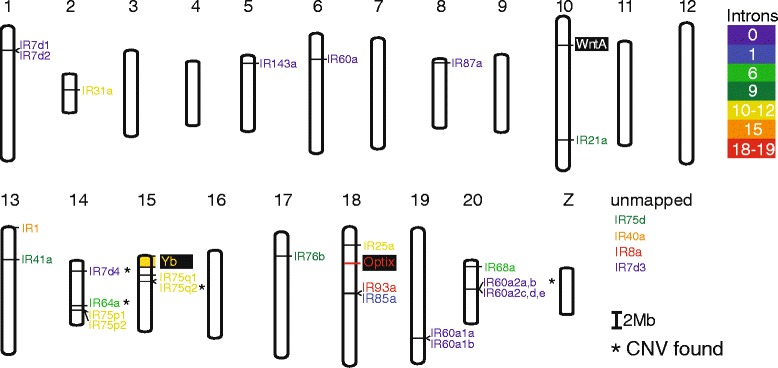
Chromosomal locations of *H. melpomene* IRs. Chromosomal location of IR genes that map to the *H. melpomene* reference genome and IRs for which the position is unknown are reported in the right corner. Different colors are used to represent the number of introns in each IR gene. Black boxes indicate the genomic location of known color pattern genes controlling black (*WntA*), yellow (*Yb*) and red (*Optix*) wing color patterns [78]. The asterisks indicate genes that display copy number variations in *H. melpomene* and/or *H. cydno*

Our IR gene models in *H. melpomene* consisted of one to 19 exons with 14 of 31 genes being intronless and only four with an elevated number of introns (≥15) (Fig. 2, Additional file 1). The IR gene models were on average 647 amino acids (AAs) in length with individual gene models ranging from 546 to 899 AAs. A genomic location for four of the 31 IRs (*IR75d, IR40a, IR8a, IR7d3*) could not be identified and thus these genes were unmapped. The remaining 27 IRs are located on 24 scaffolds, representing 13 chromosomes (Fig. 2, Additional file 1). From our *H. melpomene* genomic and transcriptomic dataset we did not find any evidence of possible pseudogenes or alternative splicing. However, we found evidence of four recent gene duplication events in the *H. melpomene* genome. These genes are *IR60a1a* and *IR60a1b* on Chromosome 19, and *IR60a2a, IR60a2b*, *IR60a2c, IR60a2d,* and *IR60a2e* on Chromosome 20 (Fig. 2). We also found evidence of older duplications shared with *B. mori* and *D. plexippus*, but not with Diptera; therefore they happened between 100 and 270 MY ago [60]. These lepidopteran duplications are *IR7d1* and *IR7d2* on Chromosome 1, *IR75p1* and *IR75p2* on Chromosome 14, and *IR75q1* and *IR75q2* on Chromosome 15 of the *H. melpomene* reference genome. While some IRs map to chromosomes containing major wing color pattern genes, none of these IR genes are tightly linked to color pattern loci (Fig. 2).

### Evolution of ionotropic receptors in Lepidoptera

Our comparative analysis between two butterflies’ (*H. melpomene*, *D. plexippus*), a moth’s (*B. mori*) and a fly’s (*D. melanogaster*) genomes allowed us to identify dipteran-, lepidopteran-, butterfly-, and *Heliconius*-specific IRs (Fig. 1). *D. melanogaster* demonstrated higher diversity, with 35 more IR genes than *H. melpomene*, mainly due to the *IR20a* clade [25], a large group with no orthologues in the three Lepidopteran species (Fig. 1, green background). Ten IR genes were Lepidoptera-specific (yellow background), namely: *IR7d1, IR7d2, IR7d3, IR143a, IR60a2, IR60a1, IR75p1, IR75p2, IR75q1*and *IR75q2* (Fig. 1). *IR7d4* was found in *H. melpomene,* and *D. plexippus* but not in *B. mori,* thus representing a butterfly-specific receptor (Fig. 1, orange background). Intriguingly, we identified two *H. melpomene*-specific IR gene expansions (*IR60a1* and *IR60a2*) (Fig. 1, red background). *IR60a1* has two copies and *IR60a2* has five copies which represent *Heliconius*-specific expansions.

### Ionotropic receptor diversity and evolution across the *Heliconius* genus

**Fig. 3:**
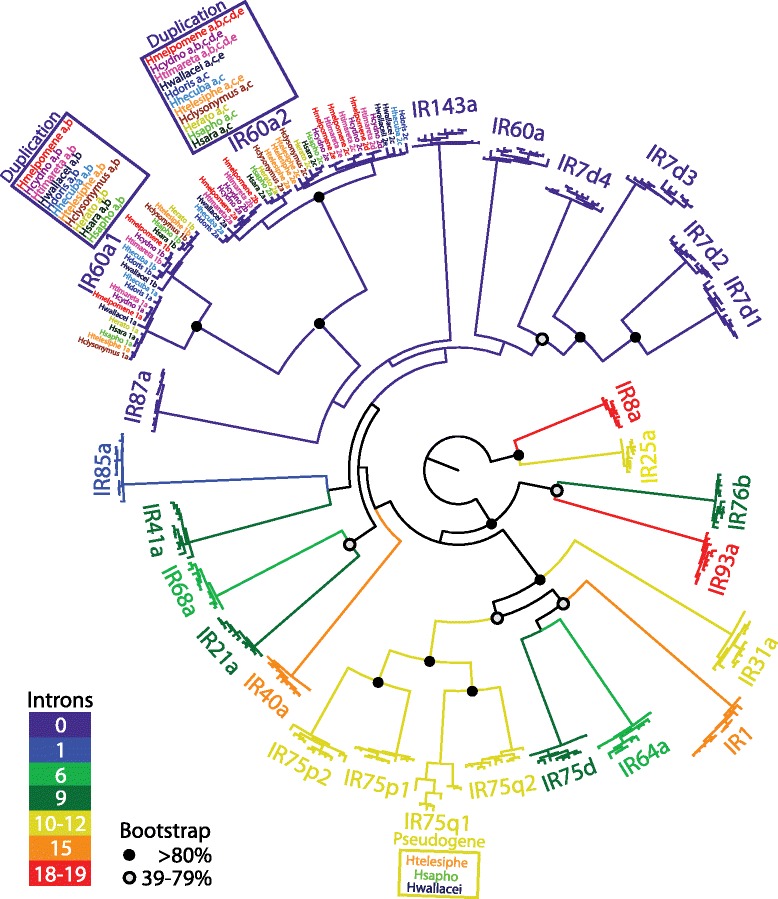
IR phylogeny in *Heliconius*. A phylogenetic relationship of the IRs across 11 *Heliconiu*s species is presented. Weak (50–79 %) and strong (80 %) bootstrap values are shown with solid and empty black dots respectively, but only for major branches. Support for branches of homologues of the 11 species that group into one gene are left out. When a gene is present in one copy for all 11 species, full gene names were left out. While the majority of the IRs are conserved between the 11 species analyzed we observed *Heliconius-*specific expansions at *IR60a1, IR60a2*, and the divergent *IR75q1* gene. Colors of each branch represent the number of exons in each specific IR gene

Comparision of 11 *Heliconius* species allowed us to study the diversity and evolution of the IRs in this genus (Fig. 3 & Additional file 3). We identified 28 out of the 31 genes in almost all 11 species. The level of conservation of these 28 genes is higher than the other two chemosensory gene families (GRs and ORs) previously analyzed by Briscoe et al. [37]. Overall, our analysis revealed a strong level of conservation in the IRs, suggesting an ancestral function.

**Fig. 4:**
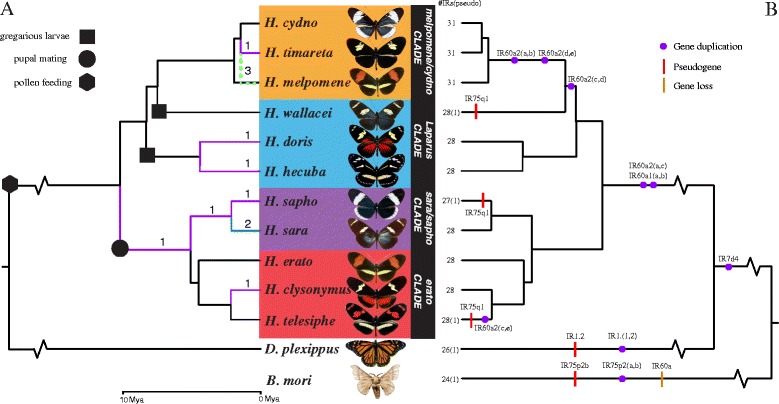
IR evolution in *Heliconius* butterflies. **a** Number of genes per branch under positive diversifying selection as determined using HyPhy’s branch-site random effects likelihood model [51]. Most positive selection maps onto the *H.melpomene*/*H. cydno* clade and *H.sara*/*H.sapho* clade. The phylogeny taken from Kozak et al. [52]. **b** Overview of gene duplication, pseudogenization and gene loss in *Heliconius*, *D. plexippus* and *B. mori*. Duplications of *IR60a1* and *IR60a2* map within the *Heliconius* genus. *IR7d4* is shared by the butterflies but not with the moth

**Fig. 5:**
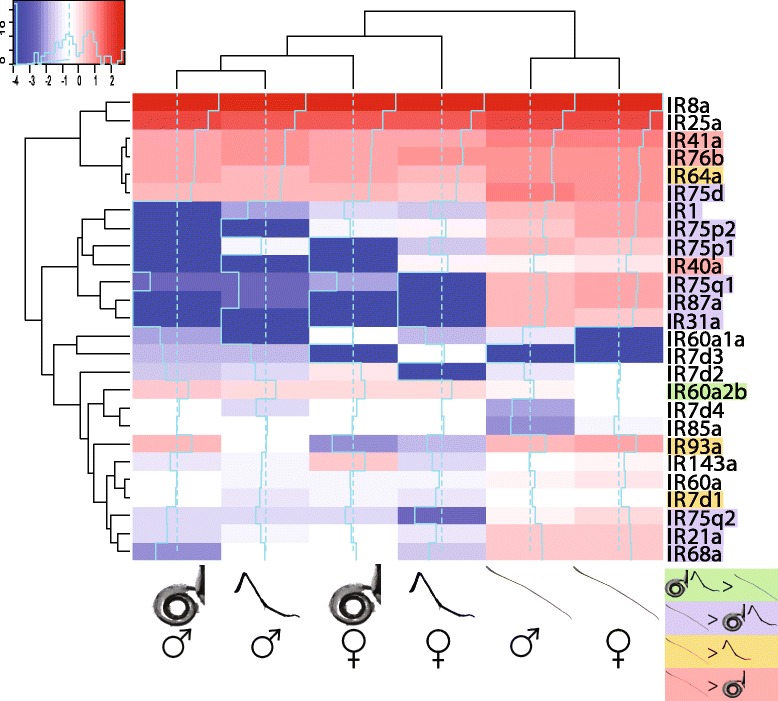
*H. melpomene* IR gene expression heatmap for sensory organs and sexes. Names of IR genes significantly differentially expressed (FDR <0.05) between mouthparts, legs and antenna are highlighted with different background colors. Most of the differentially expressed genes display higher expression in antennae than in mouthparts and legs (*purple* background). Only *IR60a2b* displayed higher expression in mouthparts and legs compared to antenna (*green* background). The remaining mRNAs that are significantly DE were more abundant in antennae than in legs (*yellow* background) or mouthparts (*red* background). Note the similarity in overall expression of legs and mouthparts compared to antennae. FPKM values are represented with a color gradient from blue to red. Exact FPKM values can be found in Table 4 and Additional file 4. Individual heatmaps are presented in the Additional file 5

Despite the broad IR gene conservation across *Heliconius*, we identified several examples of gene duplication and pseudogenization. We found that the two *H. melpomene-*specific duplications (*IR60a1* and *IR60a2*, see Fig. 1) were also duplicated in several other *Heliconius* species (Fig. 3). We found *IR60a1*a and *IR60a1b* in all 11 *Heliconius* species. While *IR60a2* had five copies *IR60a2*(*a, b, c, d* and *e*) in *H. melpomene*, *H. cydno,* and *H. timareta*, only *IR60a2* (*a, c* and *e*) were found in *H. wallacei* and *H. telesiphe* (Figs. 3 and 4b). All the other species displayed only one duplication, *IR60a2a* and *IR60a2c* (Fig. 3). The most interesting pattern emerging from the data is the presence of the two extra copies *IR60a2b* and *IR60a2d* unique to *H. melpomene*, *H. cydno,* and *H. timareta* clade (Figs. 4 and 5b). Finally, *IR75q1* was the only receptor with pseudogenes, and showed lower sequence conservation as shown by longer branch lengths in Fig. 3. Conservation among *H. wallacei*, *H. sapho,* and *H. telesiphe* was so low that in some cases the complete sequence was not traceable in the genome (Fig. 4b).

### Evidence for positive diversifying selection of ionotropic receptors within *Heliconius*

Using HyPhy [51] in Datamonkey [50] we found 12 instances of positive diversifying selection (Fig. 4a). A total of 8 IRs (*IR7d1, IR7d4, IR60a2a, IR60a1a, IR143a, IR68a, IR75d, IR93a*) had branches which show signs of positive selection (Table 2). Out of the 12 instance of positive diversifying selection, the majority was found in the *H. sapho*/*sara* clade (four out of 12), and the *H. melpomene*/*timareta* clade (four out of 12) (Fig. 4a and Table. 2)*.* Only *IR60a1a* was under positive selection on a major branch of the phylogeny, during the formation of the *H. erato*/*sapho*/*sara* clade (Fig. 4a and Table 2). Interestingly three genes, *IR143a, IR60a2a,* and *IR75d* showed signs of positive selection in *H. melpomene* and *H. timareta* but not in the species *H. cydno* (Fig. 4a and Table. 2). This resulted in a gene tree that grouped *H. timareta* with its mimic *H. melpomene* instead of *H. cydno* (Fig. 4. green dotted line). Finally, the majority of the genes (50 %) showing sign of positive selection were intronless. Only 19 % of genes with introns had at least one branch with a dN/dS >1.

**Table 2:** Branches of gene trees under positive selection as determined using HyPhy branch site random effects likelihood model [51]

Gene	Branch	Mean Ω	Ω_1_	p_1_	Ω_2_	p_2_	Ω_3_	p_3_	LRT	*p*-value	Corrected *p*-value
*IR143a*	H. timareta*H. melpomene	0.151	0	0.963	0	0.035	10000	0.002	11.309	0.000	0.008
*IR60a1a*	H. erato*H. telesiphe*H. clysonymus*H. sapho*H. sara	0.196	0.165	0.994	0.167	0.001	3333	0.005	11.042	0.000	0.009
*IR60a1a*	H. sapho*H. sara	0.178	0.151	0.983	0.149	0.013	10000	0.004	8.989	0.001	0.027
*IR60a2a*	*H. sapho*	0.434	0.055	0.942	0.163	0.026	22	0.032	19.785	0.000	0.000
*IR60a2a*	*H. sara*	0.839	0.762	0.975	0.763	0.015	199	0.010	14.949	0.000	0.001
*IR60a2a*	H. timareta*H. melpomene	0.501	0.402	0.984	0.401	0.000	5197	0.016	36.954	0.000	0.000
*IR68a*	*H. hecuba*	0.244	0.206	0.992	0.204	0.005	263	0.003	8.198	0.002	0.044
*IR75d*	H. timareta*H. melpomene	0.155	0.091	0.971	0.151	0.025	2181	0.004	14.184	0.000	0.002
*IR7d1*	*H. sara*	0.498	0.463	0.973	0.462	0.025	10000	0.002	9.936	0.001	0.017
*IR7d4*	*H. timareta*	0.079	0.033	0.994	0.032	0.002	5447	0.004	13.677	0.000	0.002
*IR93a*	*H. clysonymus*	0.131	0.079	0.991	0.080	0.005	80	0.004	12.607	0.000	0.004
*IR93a*	*H. doris*	0.287	0	0.950	0	0.001	8	0.049	10.858	0.000	0.010

IR gene name and phylogenetic branch showing positive selection is reported in the first two columns. Values obtained from HyPhy for positive selection are shown only for the IR genes supported by a significant p-value obtained from the likelihood ratio test (LRT) and corrected for multiple testing with the Holm formula

### Copy number variations in *H. melpomene* subspecies and *H. cydno*

We observed significant copy number variation only in seven out of 20 individuals analyzed (Table 3). Overall, the percentages of IRs for which CNVs are found in *H. melpomene* are similar to ORs (12.9 % vs 18.5 %, Fisher’s exact test, two-tailed, *P* = 0.57) but lower than GRs (12.9 % vs 54.4 %, Fisher’s exact test, two-tailed, *P* = 0.0001). We found evidence of CNVs for *IR75q1, IR64a, IR60a2b* and *IR7d4* in *H. melpomene*, and *IR75q2* and *IR60a2a* in *H. cydno* (Table 3)*.* Population-specific copy number variation was found in *IR60a2b* (2 *H. m. aglaope,* and 2 *H. m. amaryllis*), and in *IR7d4* (2 *H. m. melpomene*) (Table 3). All together these results suggest the possible existence of individual differences in the number of IR genes across *Heliconius* populations.

**Table 3:** Copy Number Variations (CNVs) in populations of *H. melpomene* and *H. cydno*

Species	Butterfly	Del/dup	Gene	Chromosome	Scaffold size	Begin gene	End gene	Begin CNV	End CNV	Length CNV	Norm. RD	*P*-value
*H. c. chioneus*	553	dup	*IR75q2*	Chr15	190001	51255	62232	41501	62300	20800	2.64	3.87E-08
*H. m. amaryllis*	11-160	del	*IR75q1*	Chr15	190001	67972	71764	66901	72200	5300	0.44	3.01E-11
*H. m. amaryllis*	11-160	del	*IR64a*	Chr14	16065	3076	6448	2301	5400	3100	0.5	3.50E-06
*H. m. melpomene*	9315	dup	*IR7d4*	Chr14	89318	65125	66960	55501	68600	13100	6.52	0
*H. m. melpomene*	13435	dup	*IR7d4*	Chr14	89318	65125	66960	55001	68600	13600	3.75	0
*H. m. aglaope*	09-122	del	*IR60a2b*	Chr20	72055	37488	39341	38201	39400	1200	0.23	0.0174
*H. m. aglaope*	11-569	del	*IR60a2b*	Chr20	72055	37488	39341	38201	39400	1200	0.16	0.0023
*H. m. amaryllis*	09-216	del	*IR60a2b*	Chr20	72055	37488	39341	37401	39400	2000	0.37	0.0472
*H. m. amaryllis*	11-160	del	*IR60a2b*	Chr20	72055	37488	39341	38101	39400	1300	0.2	0.0127
*H. c. chioneus*	553	dup	*IR60a2a*	Chr20	72055	47451	49370	41401	49500	8100	2.15	0

Output of CNVnator is reported here. Butterfly ID, deletion or insertion, IR gene name, chromosomal position, scaffold size, coordinates of the IR gene, coordinates and length of the CNV, normalization and *P*-value are shown. Only insertion and deletion variants with a read depth >2 (duplications) and <0.5 (deletions) are reported

### *H. melpomene* RNA-Seq expression profile of ionotropic receptor genes in mouthparts, legs, and antennae of males and females

Several IR genes showed differential expression between mouthparts, legs and antennae in males and females of *H. melpomene* (Fig. 5, Additional files 4 & 5). A total of 26 out of the 31 genes were expressed in at least one tissue type, but most genes were expressed in both sexes and all three tissues (Table 4). We did not detect expression for some *Heliconius* specific duplications, *IR60a2a, IR60a2c, IR60a2d, IR60a2e* and *IR60a1b*, which is likely due to their expression being below our detection threshold. The expression profile of the 26 detected IR genes showed strong similarity between mouthparts and legs, and a distinct expression pattern in the antennae (Fig. 5). However, within each tissue type we also observed a sex-specific expression profile (Fig. 5).

**Table 4:** *H. melpomene* IR gene expression levels between sensory tissues and sexes. Average FPKM values obtained from three biological replicates are reported for each IR gene. The false discovery rate (FDR) is provided only for the gene that is significantly differentially expressed. Only for these genes do we reported the direction of the significant comparison and the FDR value. More detailed information on FPKM values can be found in Additional files 4 & 5

IR	FPKM antenna	FPKM legs	FPKM mouthparts	FPKM average	Tissue specificity	FDR antennae vs. legs	FDR antennae vs. mouthparts	FDR legs vs. mouthparts	FDR Male vs Female
*IR60a1a*	0.02	0	0.08	0.04					
*IR7d3*	0	0.26	0.01	0.09					
*IR85a*	0.05	0.41	0.21	0.22					
*IR7d1*	0.4	0.09	0.17	0.22	ant>leg	9.86E-03			
*IR7d4*	0.11	0.12	0.43	0.22					
*IR7d2*	0.17	0.02	0.63	0.27					
*IR60a*	0.79	0.14	0.24	0.39					
*IR40a*	0.77	0.47	0	0.41	ant>mouth		5.28E-03		
*IR75q2*	1.22	0.03	0.05	0.43	ant>leg&mouth	1.41E-12	6.16E-10		
*IR143a*	0.39	0.08	1.23	0.57					
*IR21a*	2.2	0.08	0.07	0.78	ant>leg&mouth	2.49E-15	1.75E-08		
*IR68a*	2.57	0.08	0.14	0.93	ant>leg&mouth	1.13E-04	1.43E-03		
*IR31a*	3.82	0	0	1.27	ant>leg&mouth	6.65E-28	4.41E-28		
*IR75p1*	3	0.06	0	1.02	ant>leg&mouth	5.87E-09	6.11E-15		
*IR60a2b*	0.26	1.51	2.02	1.26	leg&mouth>ant	3.51E-04	1.06E-04		
*IR75p2*	4.06	0.17	0.05	1.43	ant>leg&mouth	8.98E-04	4.67E-10		
*IR1*	4.68	0.01	0.02	1.57	ant>leg&mouth	5.14E-30	2.59E-29		
*IR87a*	5.89	0	0	1.96	ant>leg&mouth	8.58E-37	4.86E-37		
*IR75q1*	6.93	0	0.01	2.31	ant>leg&mouth	5.84E-30	1.79E-30		
*IR93a*	5.4	0.15	2.23	2.59	ant>leg	7.26E-09			M>F, legs FDR = 0.032, mouthparts FDR = 1.67E-05
*IR64a*	12.44	4.72	5.89	7.68	ant>leg	1.17E-02	8.99E-02		
*IR75d*	14.77	3.16	3.81	7.24	ant>leg&mouth	9.09E-04	1.28E-04		
*IR76b*	10.77	9	6.86	8.88	ant>mouth		3.62E-03		
*IR41a*	19.5	8.46	6.47	11.47	ant>mouth		2.31E-03		
*IR25a*	75.39	44.89	57.54	59.28					
*IR8a*	734.67	462.02	474.75	557.15					

**Fig. 6:**
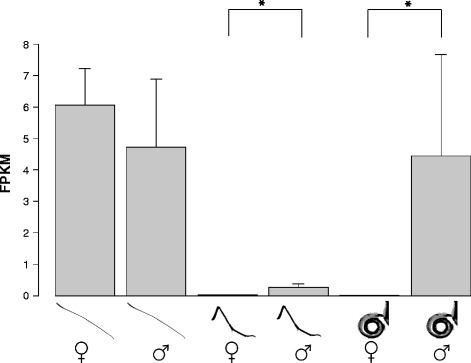
Histogram showing sex-specific gene expression of *IR93a.* Average level of *IR93a* expression (FPKM) and standard deviation for each tissue type and sex is reported. Asterisks indicate significant differential expression between male and female legs and mouthparts

From our RNA-Seq data we identified a total of six genes (*IR8a, IR25a, IR41a, IR76b, IR64a* and *IR75d*) highly expressed across all tissues and sexes. Overall, the gene expression profile indicated higher expression in the antennae than in mouthparts and legs (Fig. 5). Sixteen IRs were significantly more highly expressed in the antennae (Table 4): antennae > mouthparts and legs (*IR1, IR21a, IR31a, IR68a, IR75d, IR75p1, IR75p2, IR75q1, IR75q2* and *IR87a*) (Fig. 5, purple shade); antennae > mouthparts (*IR41a, IR76b, IR40a*) (Fig. 5, red shade); and antennae > legs (*IR64a, IR93a, IR7d1*)(Fig. 5, yellow shade). *IR60a2b* was the only gene with a gustatory expression profile, being significantly higher expressed in mouthparts and legs than antennae (Fig. 5, green shade). No gene showed a significant difference in expression between legs and mouthparts. Only one gene, *IR93a* was differentially expressed between sexes, with males showing higher expression in the legs and mouthparts than females (Table 4, Fig. 6). The remaining genes had a similar expression between the sexes, at a very low level (*IR7d2, IR7d3, IR7d4, IR60a1b, IR60a, IR85a* and *IR143a*), or a very high level (*IR8a, IR25a*). The very high expression for *IR8a* and *IR25a* was expected because they function as co-receptors [27] with *IR8a* expression about 8 times higher than *IR25a*.

## Discussion

### A complete annotation of ionotropic receptors in *Heliconius melpomene* provides novel information on IR evolution in Lepidoptera

With 31 IRs in *Heliconius* and a significantly increased number of IR genes in *Danaus* and *Bombyx,* our work suggests that Lepidoptera in general may have more IR genes than previously recognized (Table 1). Our analyses increased the number of IRs in *Danaus* and *Bombyx* [26], and significantly improved many of their gene models (Table 1). We revealed instances of taxon-specific IR duplications in *H. melpomene*, *D. plexippus*, *B. mori*, and *D. melanogaster. Drosophila* displayed by far the highest number of IRs and lineage-specific receptors. However, our data suggested that Lepidoptera have also evolved specific ionotropic receptors (Fig. 1, yellow shade). The majority of these Lepidoptera specific genes, which encompasses four large sub-families (*IR7d*, *IR60a*, *IR75p*, and *IR75q*) and a single IR locus (*IR143a*), were either described here for the first time or their annotation significantly improved with our gene models (see Table 1). We know from *Drosophila* that members of the IR7 group are involved in gustation [26], while expansion in the IR75 sub-family has been associated with the increased need to discriminate among very closely related chemicals [16]. However, it is not yet known to what extent such functions are conserved between Diptera and Lepidoptera.

On a broader scale, our data is generally in agreement with patterns observed in *Drosophila* [26] where “antennal” IRs are more conserved than “divergent” intronless IRs. In Lepidoptera however, the amount of gain and loss is less profound than in *Drosophila*, which have approximately four times as many “divergent” IRs. This pattern of diversification between “divergent” and “antennal” IRs is similar to that observed in *Heliconius* between GRs and ORs, with GRs being more diverse than ORs [37].

### Candidate IR genes of evolutionary significance across the *Heliconius* adaptive radiation

To the best of our knowledge *Heliconius* is the only other genus after *Drosophila* where the evolution of chemosensing has been explored in several species [37]. The *H. melpomene* clade displayed the largest IR repertoire (31) across the 11 *Heliconius* species analyzed (Fig. 4b). Only one pseudogene was found, *IR75q1*in *H. wallacei*, *H. sapho,* and *H. telesiphe* (Figs. 3 and 4b). However, *IR75q1* is still a potentially functional gene in other species. The reason why *IR75q1*, which is shared between butterflies and moths, is less conserved than any other IR gene (see its long branch in Fig. 3) in the *Heliconius* lineage is unknown.

Possibly the most intriguing observation that emerged from our analyses is the repeated gene duplication and evolution of *IR60a*, especially for *H. melpomene, H. cydno,* and *H. timareta,* which have two extra copies *IR60a2b* and *IR60a2d*. Interestingly, *H. melpomene, H. cydno,* and *H. timareta* are incompletely reproductively isolated, but with strong assortative mating. Moreover, *H. melpomene* and *H. timareta* are sometimes near-perfect mimics of each other [61–63]. It is likely that these species utilize specialized pheromones to distinguish among themselves [32]. The complex mating behaviors observed in *Heliconius* butterflies supports this interpretation. While the initial approach to females is strongly influenced by visual cues, close contact during courtship offers multiple opportunities for volatile and non-volatile chemical communications to occur. Thus, it is highly possible that chemical and behavioral cues may also contribute to prezygotic barriers [64]. The two extra copies*, IR60a2b* and *IR60a2d,* might be used by the *melpomene/cydno/timareta* complex to distinguish chemical cues during mate choice.

### Evidence for positive selection of ionotropic receptors within *Heliconius*

The ratio of substitution at non-synonymous (dN) versus synonymous (dS) sites has been widely utilized to quantify evolutionary pressures on proteins [65]. Despite limitations and controversies over the model used to evaluate gene evolution [66, 67], the dN/dS ratio is a widely used approach to quantify selection pressures acting on protein-coding regions, owing in part to its simplicity and robustness. Our results are based on only one sequence per species, because dN/dS is not designed for within population comparison [67]. Our analysis did not take in consideration instances of population and species-specific nucleotide variation.

We found the majority of the genes with elevated dN/dS in the two lineages represented by *H. sara* and *H. sapho* (*IR60a1a*, *IR60a2a*, and *IR7d1*) and *H. melpomene* and *H.timareta* (*IR143a*, *IR60a2a,* and *IR75d*) (Fig. 4a). Interestingly, *H. sapho* and *H. sara* are unusual in that they do not synthesize, but instead sequester most of their toxic cyanide compounds from their host plants and therefore specialize on only a few species of *Passiflora* [19]. It is therefore possible that these receptors represent candidate genes for host-plants recognition. Conversely, *H. melpomene* and *H. timareta* are more generalists, but represent a clade of very young species which are still not reproductively isolated [16, 17, 31]. Thus, the three receptors under positive selection in these two species (*IR143a, IR60a2a*, and *IR75d*) might be part of a toolkit of chemosensory genes that contributes to the establishment of prezygotic isolation based on chemical cues. In contrast, we found very few IRs with elevated dN/dS in the *erato* clade (*H. erato, H. clysonymus*, and *H. telesiphe*), and the *Laparus* clade (*H. wallacei*, *H. doris*, and *H. hecuba*). Lastly, only *IR60a1a* had a significantly elevated dN/dS at an older phylogenetic node comprising several distinct clades (Fig. 3 and Table 2). This phylogenetic node represents the ancestor of the pupal-maters, a group of butterflies that share a unique behavior, which consists of males being able to localize and mate with uneclosed or with freshly emerged females [68].

### Copy number variation in natural populations of *H. melpomene* and *H. cydno*

Copy number variations (CNVs) are now recognized as important components of genomic diversity, alongside single nucleotide polymorphisms (SNPs) [69]. However, few studies have explored CNVs in chemosensory gene families of non-human populations [70, 71]. To date, only one study has explored CNVs in chemical receptor families across a butterfly genome [37]. This study of *H. melpomene* found widespread CNVs in GRs and ORs, with CNVs more frequent among GRs than ORs [37]. Our data suggest a modest number of *H. melpomene* IR genes with CNVs, comparable to their ORs [37]. Intriguingly, half of the CNVs occur in *IR60a2b*, one of two receptors uniquely found in *H. melpomene*, *H. cydno* and *H. timareta* and the only gene with higher expression in legs and mouthparts than in antennae (Figs. 3, 4 and 5). *IR60a2b* showed reduced read depth in the populations of *H. m. aglaope* and *H. m. amaryllis* (Table 3). Although these CNVs might be involved in the evolution of ecologically relevant traits, further work will be needed to elucidate their functional significance, if any.

### RNA-Seq provides insights into tissue- and sex-specific IR expression in the sensory organs of *H. melpomene*

Very little is known about the expression patterns of ionotropic receptors. In *D. melanogaster* IR genes are not only expressed in antennae [16] but also in the labellum, legs, pharynx, and anterior wing margin [25], thus suggesting a more complex function for this receptor family than previously envisioned. The majority of the *H. melpomene* IRs were more highly expressed in antennae compared to mouth parts and legs (Fig. 5, Additional files 4 & 5). In accordance with previous studies, the two co-receptors, *IR8a* and *IR25a*, have the highest and most homogeneous expression level across all tissues and sexes [72, 73]. However, *IR8a* is expressed ~eight times more than *IR25a,* which contrasts with *Drosophila,* where the two genes have similar expression levels [16]*,* and with *Aedes aegypti* where *IR25a* is expressed tenfold more than *IR8a* [72]. Only in *Culex quinquefasciatus* is the expression of *IR8a* and *IR25a* similar to that in *Heliconius* [73]. These differences in gene expression at *IR25a* and *IR8a* could be driven by selection for expression levels of other chemical-specific receptors,for example, the *IR20a* clade is mostly expressed together with *IR25a* [16, 74], which implies that chemical-specific IRs differ significantly between species. In addition to *IR8a* and *IR25a,* we also identified four additional genes (*IR41a*, *IR76b*, *IR64a* and *IR75d*) with elevated expression across tissue types and sexes. All these genes display a strong sequence homology between Diptera and Lepidoptera. Although no specific function is known in *Heliconius*, studies in *Drosophila* have shown the involvement of *IR41a* and *IR64a* in amino-sensing: specifically *IR41a* is sensitive to 1,4-diaminobutane [75], while *IR64a* is sensitive to acetate, propionate and butyrate [76]. Moreover, in *D. melanogaster IR76b* has been shown to be co-expressed with other IRs suggesting that it might act as a second type of co-receptor [16, 74]. The ubiquitous and high expression of *IR76b* in *H. melpomene* seems to support this theory.

Like *D. melanogaster* [27], our analysis identified several genes that are significantly higher expressed in antennae than in legs or mouthparts (Fig. 5, Table 4, Additional files 4 & 5). This translates into the antennae differing from legs and mouthparts in their overall expression profile (Fig. 5). Such clustering likely represents the different chemosensing functions of these tissues, with mouthparts and legs being utilized in tasting while antennae are more attuned for smelling [16, 25]. Only one gene, *IR60a2b*, was significantly higher expressed in legs and mouthparts compared to antennae (Fig. 5, Table 4, Additional files 4 & 5), suggesting a gustatory function, possibly related to host plant recognition or mate choice. Our heat map groups male legs with male mouthparts and vice versa for females, which suggests a distinct ability of males and females to perceive compounds (Fig. 5, Additional file 5). However, only *IR93a* was differentially expressed between sexes but only in legs and mouth parts (Table 4, Figs. 5 and 6). Unfortunately nothing is known of the function of these two promising candidate genes, *IR60a2b* and *IR93a*.

## Conclusions

Although *Heliconius* are best known for their stunning visual signals and vision far into the ultraviolet [77], our work and the recent study of Briscoe et al. [37] have shown an elaborate chemosensory system consisting of ~165 chemosensory receptors. Our work characterized the least studied chemosensory gene family in Lepidoptera, the ionotropic receptors (IRs). We identified instances of IR duplication and pseudogenization in Lepidoptera, butterflies, and within *Heliconius*. The most notable butterfly-specific IR gene duplications are of *IR7d4*, and of *IR60a1* and *IR60a2*, which are *Heliconius-*specific. Among these *Heliconius* duplications, *IR60a2b* was uniquely found in the incompletely reproductively isolated species, *H. melpomene*, *H. cydno,* and *H. timareta*, and was the only gene significantly more expressed in legs and mouthparts than in antennae. Moreover, two additional genes that should be mentioned for their unique characteristics are: *IR60a1*, which displayed an elevated d*N*/d*S* in a major phylogenetic branch encompassing several species associated with the evolution of pupal mating, and *IR93a* that was the only gene with sex specific expression. Overall our work has generated a list of *Heliconius* candidate IR genes of evolutionary significance, which could have important implications for their chemical-mediated behaviors and ecological adaptations leading to speciation. Unfortunately functional studies of chemosensory genes are still absent in Lepidoptera, thus hampering our understanding of the specific roles of these genes. As we continue to learn more about their function, our understanding of the link between these receptors, butterfly behaviors and the evolution of relevant ecological traits will greatly improve.

## Availability of supporting data

Raw data for *Heliconius cydno* (ERS235661), *Heliconius doris* (ERS977668), *Heliconius hecuba* (ERS977683), *Heliconius timareta* (ERS977723), *H. sara* (ERP002444), *H. sapho* (ERP002444)*, Heliconius clysonymus* (ERS1061651) and *Heliconius telesiphe* (ERS1061652) are available at the European nucleotide archive.

All *H. melpomene* IR genes (KU702609-KU702639), and the intronless genes for the other 10 *Heliconius* species (KU756934 - KU757038) are available on genbank.

## Additional files

Additional file 1: Excel spreadsheet with *H. melpomene* IRs with their genomic location and intron- and exon- boundaries. (XLSX 37 kb) Additional file 2: Fasta file for all manually curated IRs in the three lepidopterans: *Bombyx mori*, *Danaus plexippus* and *Heliconius melpomene*. (TXT 155 kb) Additional file 3: Fasta file for all manually curated IRs in the 11 *Heliconius* species utilized in this study. (TXT 610 kb) Additional file 4: Excel spreadsheet with all detailed FPKM expression values. (XLSX 13 kb) Additional file 5: Heatmap of IR gene expression for each *H. melpomene* biological replicate utilized in this study. Exact FPKM numbers can be found in Table 4 and Additional file 4. (EPS 8200 kb)

## Abbreviations

IR: ionotropic receptor
GR: gustatory receptor
OR: olfactory receptor
